# Right Ventricular Outflow Tract Melanoma Diagnosed Using a Transfemoral Biopsy Through a Shortened Jr-4 Guiding Catheter

**DOI:** 10.1016/j.cjco.2023.09.002

**Published:** 2023-09-12

**Authors:** Arnaud Planchat, Nana K. Poku, Claudio De Vito, Jean-François Deux, Stéphane Noble

**Affiliations:** a Department of Medicine, Cardiology Division, Structural Heart Unit, Geneva University Hospitals, Geneva, Switzerland; b Department of Diagnosis, Clinical Pathology Division, Geneva University Hospitals, Geneva, Switzerland; c Department of Diagnosis, Radiology Division, Geneva University Hospitals, Geneva, Switzerland

**Figure 1:**
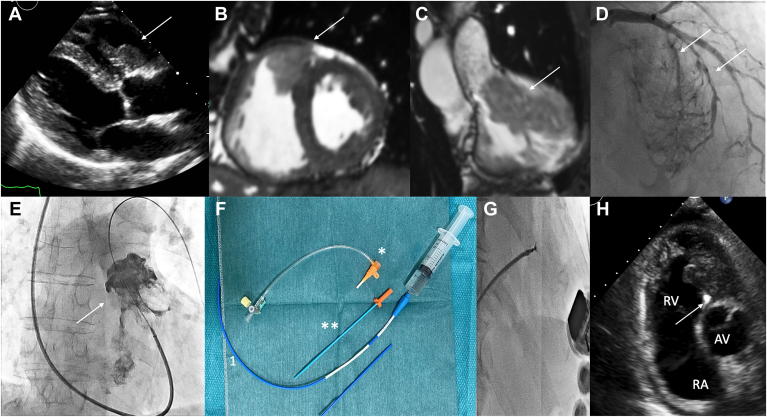
(**A**) Transthoracic echocardiography (TTE), end-diastolic parasternal long-axis view, **arrow** indicating right ventricular mass. (**B**) Axial short-axis cine magnetic resonance imaging, **arrow** indicating right ventricular outflow tract (RVOT) mass. (**C**) Sagittal cine magnetic resonance imaging, right ventricular outflow, **arrow** indicating RVOT mass. (**D**) Coronary angiography, left anterior oblique cranial, **arrows** indicating vascularization of the mass by 2 septal branches. (**E**) Angiography of the mass through a right internal jugular access, **arrow** indicating the RVOT pediculated mass. (**F**) 8-F (**blue**) 100-cm-long right coronary Judkins 4 Launcher (Medtronic) guiding catheter, which was shortened by cutting approximately 15 cm (shown by 1). We reconnected the distal and proximal portions of the guiding catheter using the distal part of a 7-F (**orange**) Terumo (Tokyo, Japan) introducer sheath Therefore, the proximal portion of the introducer sheath with the valve was cut, (indicated by **asterisk**). The dilator (indicated by **double asterisk**) of the introducer sheath was used to facilitate the reconnection of the 2 pieces of the guiding catheter using a portion of the introducer sheath (**X**). (**G**) Biopsy guided by fluoroscopy and TTE through a right femoral access using an 8-F regular 100-cm-long guiding catheter, which was initially too long to open a 104-cm-long Cordis bioptome. (**H**) TTE, parasternal short-axis view, **arrow** indicating the bioptome (hyperechoic) collecting pieces of the RVOT mass. AV, aortic valve; RA, right atrium; RV, right ventricle.

An 83-year-old man, known for having a melanoma of the right eye choroid, treated with proton therapy in 1998, was referred 21 years later for investigation of an asymptomatic heart murmur. Transthoracic echocardiography showed a 50 × 22 mm mass in the right ventricular outflow tract, with moderate obstruction and a mean/maximum gradient of 16/23 mm Hg (Fig. 1A). Magnetic resonance imaging confirmed the presence of a pediculated hypervascular, mobile, subpulmonary mass originating from the upper interventricular septum (Fig. 1, B and C). Hyperintense character on T1 suggested the presence of melanin. Thoraco-abdominal computed tomography revealed a left upper-lobe pulmonary nodule, a right lower-lobe nodule, and a retroperitoneal mass, all hypermetabolic on ^18^F-fluorodeoxyglucose–positron-emission tomography. Coronary angiography showed vascularization of the mass by 2 septal arteries (Fig. 1D).

A multidisciplinary panel recommended biopsy of the cardiac mass, considering its accessibility. The fluoroscopy-guided procedure was performed through right internal jugular vein access (Fig. 1E) and was complicated by pericardial effusion requiring pericardiocentesis secondary to a highly mobile mass and lack of support. Histology remained inconclusive. Another biopsy guided by fluoroscopy and transthoracic echocardiography, through femoral access, was performed using an 8-F 100-cm-long right coronary Judkins 4 Launcher Medtronic (Mineapolis, MN) guiding catheter, which was too long to open the 7-F 104-cm-long Cordis bioptome. The 8-F right coronary JR4 guide (Medtronic) catheter was shortened using a 7-F terumo sheath in order to use the bioptome (Fig. 1F) and collect 4 pieces of the mass (Fig. 1, G and H). The femoral approach with the shortened JR-4 guide catheter allowed greater stability and facilitated manipulation and orientation of the bioptome to the mass without complications.

**Figure 2:**
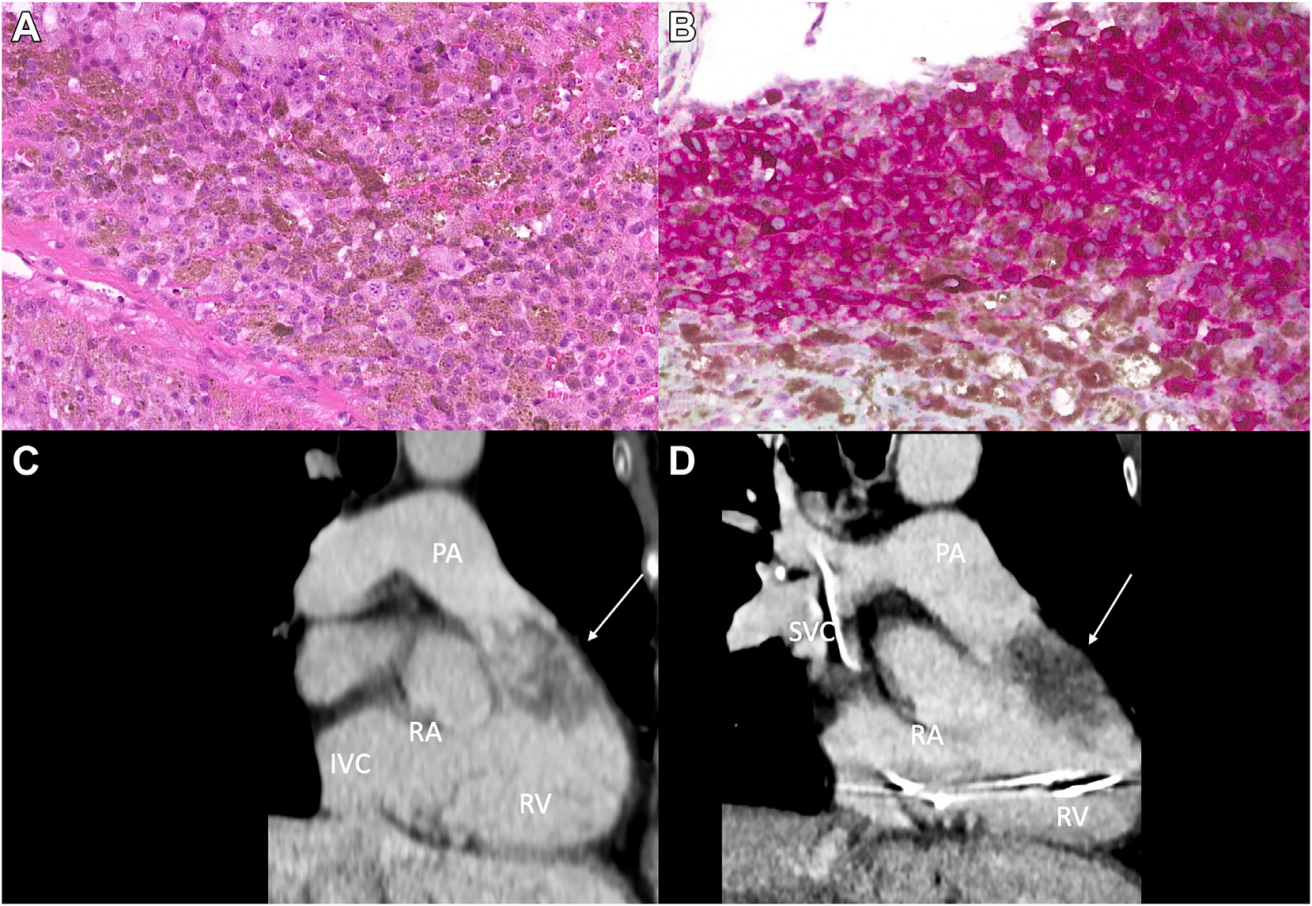
(**A**) Epithelioid cell with prominent nucleoli and pigmented cytoplasm (hematoxylin and eosin stain, ×200). (**B**) Melan A immunostaining (×200). (**C**) Right ventricular outflow track (RVOT) computed tomography scan before palliative radiotherapy. (**D**) RVOT computed tomography scan after palliative radiotherapy (**arrow** indicating dual-chamber pacemaker that was implanted in between for a complete atrioventricular block), showing stability of the cardiac mass (indicated by **arrows**) despite palliative debulking radiotherapy. IVC, inferior vena cava; PA, pulmonary artery;.RA, right atrium; RV, right ventricle; SVC, superior vena cava.

Histology analysis revealed epithelioid cells with prominent nucleoli and pigmented cytoplasm (Fig. 2A). The presence of melan A was confirmed by immunostaining (Fig. 2B), which is consistent with cardiac metastasis from a malignant melanoma. Sequencing of a 400-gene panel showed a *GNAQ G464V* and a rare *BRAF Q209L* mutation, compatible with recurrence of a uveal melanoma. Due to the obstructive nature of the mass, without any reasonable surgical option or proven systemic therapy, a targeted palliative debulking radiotherapy consisting of 48 Gy was completed. At 1 month, a computed tomography scan showed stability of the cardiac mass (Fig. 2, C and D) but progression of other lesions in number and size. The patient died 3 years later.

Even if the combination of clinical and multimodal imaging approaches can avoid a biopsy, histopathologic analysis obtained by it remains the gold standard for the diagnosis of cardiac masses. Secondary cardiac tumours are 40 times more common than primary tumours.^1^ They confer a particularly poor prognosis (1-year mortality > 50%), generally requiring palliative treatment.^2^ In melanoma, cardiac metastasis occurs in < 2% of patients with metastatic disease.^3^ Uveal melanomas are a rare subtype that responds less to immunotherapy, with response rates between 3% and 8%. Dual immunotherapy with ipilimumab and nivolumab has shown greater response but with significant toxicity.^4^ In addition, the patient had an atypical *BRAF* mutation associated with decreased sensitivity to dual anti-*MEK* and anti-*BRAF* inhibition. Given the lack of proven systemic therapies, the approach consisted of clinical and radiologic follow-up in this asymptomatic patient.

**Novel Teaching Points**

• Histopathologic analysis obtained by a biopsy is the gold standard for the diagnosis of a cardiac mass.
• Biopsy via femoral access is an option when the traditional jugular vein access presents difficulties because of the angle, location, or mobility of the mass.
• Guidance by echocardiography can increase biopsy success rates and maximize its diagnostic yield.
• Secondary cardiac tumours are more common than primary ones and are associated with a particularly poor prognosis.
